# Variations on fetal heart rate variability

**DOI:** 10.1113/JP270717

**Published:** 2016-02-29

**Authors:** C. J. Shaw, C. C. Lees, D. A. Giussani

**Affiliations:** ^1^ Department of Physiology, Development and NeuroscienceUniversity of CambridgeCambridgeUK; ^2^ Institute of Reproductive and Developmental BiologyImperial College LondonLondonUK

Intrapartum electronic fetal monitoring (EFM) is widely used throughout the developed and developing world. This practice is based on the understanding that fetal heart rate (FHR) is sensitive to hypoxia. During labour, the fetus may be exposed to acute hypoxia during uterine contractions or intrapartum insults, and has well‐established cardiovascular compensatory defence mechanisms, which ideally prevent hypoxic–ischaemic encephalopathy by maintaining perfusion pressure and substrate delivery to essential organs (Giussani *et al*. 1993). Failure of these compensatory mechanisms results in a progressively worsening metabolic acidosis. Development of severe acidaemia (pH < 7.05) is a key turning point after which fetuses are unable to maintain fetal cardiovascular defence mechanisms and cerebral perfusion, rendering them at risk of asphyxial brain injury (Gunn & Bennet, 2009). Therefore, intrapartum EFM aims to identify, via changes in FHR patterns, fetuses unable to initiate or maintain such compensatory adaptations in response to hypoxia, which become acidotic, and deliver them before they are at risk of asphyxia, severe acidosis, cardiovascular collapse, end organ damage or death.

Retrospective analysis of intrapartum EFM records shows that reduced or absent FHR variability with decelerations is the most consistent predictor of newborn acidaemia and poor outcome (Parer & Ikeda, 2007). However, the fetal physiology underlying these changes is far from clear, as are the changes in FHR patterns and variability preceding this critical end‐stage. While knowledge of both fetal physiology and the control of heart rate variability in the adult has advanced greatly in the last 50 years since intrapartum EFM was introduced, little of it has translated into uncontested interpretation of FHR patterns. It is clear that without understanding the physiology underlying the control of FHR and its variability in normal and complicated pregnancy, we will not move forwards from retrospective analysis to prospective prediction of abnormalities in the control of FHR and how these can be detected by intrapartum EFM. Such a change in approach is long overdue. A recent Cochrane review of 13 randomised controlled trials concluded that intrapartum EFM has not reduced the rates of fetal mortality or cerebral palsy and it has increased the rates of obstetric intervention in labour, caesarean section, operative vaginal delivery and delivery of non‐acidotic babies (Alfirevic *et al*. 2013). Invasive adjuncts to EFM monitoring, such as fetal blood sampling, as well as fetal pulse oximetry and fetal electrocardiography (measured as the T/QRS ratio) have all been applied with limited success in reducing operative delivery and without significant improvement in neonatal outcome (East *et al*. 2014). Guidelines and recommendations for the interpretation of intrapartum EFM exist, such as the rule‐based category colour‐coded FHR management framework championed by Parer & Ikeda (2007). Adoption of such frameworks in a single centre with dedicated training reduced the number of babies born severely acidotic without altering the rates of operative or instrumental delivery (Katsuragi *et al*. 2014). However, despite a general move towards standardised assessment and the training of clinicians to interpret intrapartum EFM with reference to such guidelines, there remains a well‐acknowledged poor reliability of EFM assessment between clinicians (Blackwell *et al*. 2011). The chronically instrumented fetal sheep preparation in late gestation, which is uncomplicated by general or local anaesthesia and in which studies follow full post‐surgical recovery, provides an incomparable window into fetal physiology that is inaccessible in the human fetus. Not only can we control the exact nature of the fetal insult in terms of magnitude, duration and frequency, but the preparation permits detailed fetal cardiovascular function analysis over prolonged periods, which can be correlated to fetal endocrine as well as blood gas, metabolic and acid/base status. Using this experimental model, previous studies have shown that acute hypoxia activates both arms of the autonomic nervous system controlling FHR but in favour of vagal dominance and that activation of the sympathetic nervous system (SNS) is key to providing rapid support to increase fetal peripheral vascular resistance and maintain blood pressure despite slowing of the fetal heart rate (Giussani *et al*. 1993). Further, advancing gestational age (Fletcher *et al*. 2006) and antenatal glucocorticoid therapy (Jellyman *et al*. 2005) affect the magnitude and pattern of the FHR response to hypoxia and of the fetal endocrine and metabolic compensation, all of which contribute to alterations in FHR variability. Surprisingly, despite their physiological and clinical importance, how gestational age or fetal glucocorticoid status affects FHR variability and how the mechanisms mediating FHR variability during acute hypoxia are altered in the chronically hypoxic IUGR fetus with or without antenatal glucocorticoid therapy still remain to be elucidated.

Increasing the body of knowledge of the physiology underlying the control of FHR variability, an interesting study using the chronically instrumented fetal ovine preparation by Lear and colleagues in this issue of *The Journal of Physiology* challenges the long held assumption that the sympathetic nervous system (SNS) mediates FHR variability in labour. This finding directly contradicts the long held clinical interpretation that preserved FHR variability implies adequate fetal physiological compensation. In this study, analysis of fetal sheep which had been chemically sympathectomised showed that FHR variability between episodes of brief repeated asphyxia or elevation of the ST segment during asphyxia was unaltered relative to sham control fetal sheep. Rather, the data imply that the predominant mediator of increased FHR variability between episodes of brief intermittent asphyxia was increased parasympathetic activity. Therefore, if the SNS does not influence FHR variability between simulated contractions, interpretation of FHR variability is unlikely to help monitor changes in SNS activity during labour, a significant change from traditional clinical understanding. Perhaps it is time to re‐examine the maxim, now 30 years old and the basis of many obstetric guidelines, that the fetus with a normal FHR variability is at a low risk of immediate death or brain injury caused by asphyxia, regardless of the presence of decelerations or bradycardia. There is an urgent need to design experiments to address the underlying physiology mediating alterations in FHR variability in healthy and complicated pregnancy at different stages of gestation, with and without exposure to common antenatal therapies, such as treatment with steroids. There is also the possibility that the fetal heart may show intrinsic variability in its heart rate, independent of autonomic innervation, which can be altered by gestational age, medications or the quality of the intrauterine environment. Once obtained, this information should be rapidly translated into frameworks for intrapartum EFM interpretation, either by clinicians or, more reliably, by a computer algorithm. Only then will basic and clinical science act in tandem to begin to reliably identify the fetus at risk of decompensating, rather than the already decompensated fetus.

## Additional information

### Competing interests

None.

### Funding

D.G. is Professor of Cardiovascular Physiology and Medicine at the Department of Physiology, Development and Neuroscience at the University of Cambridge, Professorial Fellow and Director of Studies in Medicine at Gonville & Caius College, Cambridge, a Lister Institute Fellow and a Royal Society Wolfson Research Merit Award Holder. He is supported by the British Heart Foundation RG/11/16/29260. C.C.L. is supported by the National Institute for Health Research (NIHR) Biomedical Research Centre based at Imperial College Healthcare NHS Trust and Imperial College London.
